# Genomics and proteomics of the liver fluke Opisthorchis felineus

**DOI:** 10.18699/VJ20.44-o

**Published:** 2020-07

**Authors:** V.A. Mordvinov, N.I. Ershov, O.G. Zaparina, M.Y. Pakharukova

**Affiliations:** Institute of Cytology and Genetics of Siberian Branch of the Russian Academy of Sciences, Novosibirsk, Russia; Institute of Cytology and Genetics of Siberian Branch of the Russian Academy of Sciences, Novosibirsk, Russia; Institute of Cytology and Genetics of Siberian Branch of the Russian Academy of Sciences, Novosibirsk, Russia; Institute of Cytology and Genetics of Siberian Branch of the Russian Academy of Sciences, Novosibirsk, Russia

**Keywords:** genomics, trematodes, Opisthorchis felineus, trans-splicing, microintrones, proteomics, operons, gene expression, геномика, трематоды, Opisthorchis felineus, транс-сплайсинг, микроинтроны, протеомика, опероны, экспрессия генов

## Abstract

The causative agent of opisthorchiasis, the liver fluke Opisthorchis felineus (Rivolta, 1884) is one of the helminths of humans and animals in Russia. Together with closely related species of trematodes O. viverrini (Poirier, 1886) and Clonorchis sinensis (Loos, 1907), O. felineus is a part of a triad of epidemiologically important trematodes in the family Opisthorchiidae. Adult O. felineus worms infest the hepatobiliary system of warm-blooded animals and might provoke the development of severe pathologies, including malignancy of bile duct epithelium. The high medical importance of O. felineus attracts the attention of researchers. This review briefly summarizes the data about O. felineus genomics and proteomics. The review provides a comparative analysis of the number of genes and sizes of nuclear genomes of a number of flatworms, the distribution of intron lengths, as well as results of synteny between the O. felineus, O. viverrini and C. sinensis genomes. Special attention is paid to a particular form of RNA processing known as trans-splicing, widely presented in the opisthorchiid genomes. We also provide the results of a comparative analysis of the xenobiotic metabolizing system between parasitic and free-living flatworms. Moreover, data on parasitic granulins, which are potential promoters of cholangiocyte neoplasia, are also presented. Data on the O. felineus genomics and proteomics provide first insights into the structural and functional organization of the genome of this parasitic flatworm with a complex life cycle as well as provide a significant contribution to our understanding of “host-parasite” interaction and evolution of this group of parasitic flatworms.

## Abstract (ru)

Возбудитель описторхоза, печеночный сосальщик Opisthorchis felineus (Rivolta, 1884) – один из наиболее распространенных видов гельминтов человека и животных на территории России. Вместе с близкородственными видами печеночных трематод O. viverrini (Poirier, 1886) и Clonorchis sinensis (Loos, 1907), ареалы которых расположены в Юго-Восточной Азии и на Дальнем Востоке, О. felineus составляет триаду эпидемиологически значимых трематод семейства Opisthorchiidae. Половозрелые особи (мариты) O. felineus паразитируют в гепатобилиарной системе теплокровных и при длительной инвазии провоцируют развитие тяжелых осложнений, включая малигнизацию эпителия желчных протоков. Высокая медицинская значимость O. felineus привлекает внимание исследователей, работающих в различных областях биологии и медицины. Так, в последнее время активно проводятся исследования молекулярной биологии этого представителя паразитических плоских червей. В настоящем обзоре кратко суммированы результаты исследований геномики и протеомики O. felineus, являющиеся, на наш взгляд, существенным вкладом в решение вопросов структурно-функциональной организации геномов многоклеточных паразитов со сложным жизненным циклом и изучение молекулярных механизмов взаимодействия паразит–хозяин. Приведены сравнительные данные по количеству генов и размерам ядерных геномов ряда плоских червей, распределению длин интронов, а также анализу синтении геномов описторхид O. felineus, O. viverrini и C. sinensis. Отдельное внимание уделено обсуждению особой формы процессинга РНК, известной как транс-сплайсинг, широко представленной в геноме O. felineus. В статье приводятся анализ литературных данных по генам системы метаболизма ксенобиотиков, сравнению представленности генов этой систе- мы между различными видами паразитических и свободноживущих плоских червей и обзор литературы по наличию и уровню экспрессии генов гранулинов – потенциальных промоторов неоплазии холангиоцитов. Данные по геномике и протеомике O. felineus пополняют базу знаний, обеспечивающую исследо- вание проблем эволюции трематод, патогенеза печеночных гельминтозов и гельминт-ассоциированного канцерогенеза.

## Introduction

In 1884, the Italian scientist S. Rivolta described a new species of helminths Distomum felineum (synonym Opisthorchis felineus, D. sibiricum – a liver fluke, a Siberian fluke), extracted from the bile ducts of the cat’s liver. In 1891, professor of Tomsk University K.N. Vinogradov discovered this species of liver trematodes in humans (Pozio et al., 2013).

The liver fluke O. felineus has a complex life cycle with alternating two intermediate and one definitive hosts. The list of the definitive hosts for this parasite consists of 33 species and subspecies of mammals, primarily from the Order Carnivora (carnivores): domestic cats, dogs, wolves, foxes, bears, badgers. A man is also susceptible to the infection of O. felineus (Beer, 2005).

Infection of animals and humans occurs as a result of eating raw or undercooked fish infected with metacercaria of O. felineus. After entering the digestive tract of a definitive host, the metacercaria cyst is destroyed and newly excised juvenile worm moves to the bile ducts of the liver. Upon reaching maturation, the parasites produce lots of eggs containing miracidia – the invasive life stage for the first intermediate host, Bithyniidae mollusks. Eggs pass out with the feces of mammals, enter the water reservoirs where they are ingested by the mollusks to enter and develop in. Sporocysts, redia, and cercaria – life stages of fluke with asexual reproduction are successively passed in the Bithyniidae mollusk. Free-swimming cercariae leave the mollusks and are able to infect the second intermediate host, the cyprinid fish. In fish, cercaria is encapsulated and transformed into metacercaria – the only infectious life stage for the infection of fish-eating mammals.

In humans, the infection of O. felineus, opisthorchiasis, is a long lasting disease, occurs with the exacerbations and might contribute to the development of primary liver cancer. Opisthorchiasis refers to natural focal diseases. The most indicative endemic area is the West Siberian Lowland – one of the largest lowland plains in the world. There is the world’s largest outbreak of opisthorchiasis caused by the O. felineus in the Ob-Irtysh basin (Pakharukova, Mordvinov, 2016).

In addition to Western Siberia, the range of O. felineus infection also extends to Eastern, Western and Southern Europe. This species of helminths was found in central Russia, Belarus and Ukraine, in the Baltic countries, in Germany (Schuster et al., 1999), Italy (Pozio et al., 2013), on the Balkan and Iberian peninsulas (Petney et al., 2013). According to preliminary estimates, at least 1.6 million people in the world are infected with O. felineus (Keiser, Utzinger, 2009). In the Russian Federation, up to 40 thousand cases of opisthorchiasis are detected annually (Rospotrebnadzor…, 2015). Nevertheless, these data most likely do not reflect the actual rate of the infection. The first stages of the disease and the transition to the chronic stage might pass unnoticed, and gradually appearing symptoms do not have specificity. As a result, the true number of patients with opisthorchiasis can significantly exceed the official statistics.

Existing opisthorchiasis therapy does not guarantee complete relief and does not prevent re-infection. In addition, chemotherapy for this disease has side effects and might have negative consequences for patients. In this regard, the issue of the possibility of creating new effective and safe anthelmintic agents for the treatment of opisthorchiasis is very relevant. A thorough study of the molecular biology of O. felineus liver fluke provides a key to understand the molecular mechanisms of the host–parasite interaction and to identify potential pharmacological targets for the treatment of opisthorchiasis.

This review is devoted to the research of genomics and proteomics of O. felineus, which makes a significant contribution to solving the fundamental problems of molecular parasitology and genetics, as well as the development of new approaches to facilitate diagnosis, prevention and treatment of opisthorchiasis infection.

## Genomics of Opisthorchis felineus

**Nuclear genome**

The size of the existing assembly of the nuclear genome of O. felineus liver fluke is 684 million base pairs, 30.3 % of the genome is represented by repeating elements, mainly retrotransposons. According to these characteristics, the O. felineus genome is very close to the genomes of two other epidemiologically significant species of the Opisthorchiidae family, other liver flukes O. viverrini and Clonorchis sinensis. The O. felineus genome differs significantly from the genomes of the Schistosomatidae and Fasciolidae trematodes (Table 1). There are 11,455 annotated protein-coding genes in O. felineus genome (Ershov et al., 2019), as well as 55 genes encoding microRNAs (Ovchinnikov et al., 2015). The total number of O. felineus genes is almost a third less than that for O. viverrini and C. sinensis and almost coincides with the number of S. mansoni and F. hepatica genes.

**Table 1.**
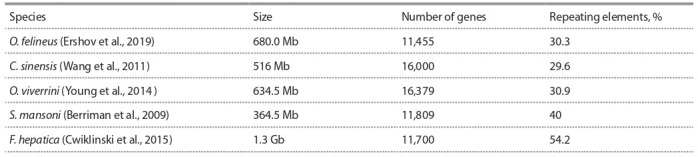
Characteristics of the genomes of five species of trematodes

Significant structural variability was found when the genomic synteny of opisthorchids O. felineus, O. viverrini, and C. sinensis was analyzed. According to the localization of homologous loci, the degree of similarity between the O. felineus and C. sinensis genomes is higher than that between O. viverrini with O. felineus (Ershov et al., 2019). These data correlate well with the results of karyotype analysis: O. felineus and C. sinensis have seven pairs of chromosomes, O. viverrini has six pairs of chromosomes (Zadesenets et al., 2012).

The data on genome synteny also align well with the results of phylogeny using separate genetic markers and genome-wide data from three species of opisthorchids. The results of these studies indicate that C. sinensis belongs to the genus Opisthorchis and do not support the isolation of this species into a separate genus Clonorchis (Shekhovtsov et al., 2009; Cai et al., 2012; Pomaznoy et al., 2016; Ershov et al., 2019). Thus, according to the findings of molecular biological studies, the taxonomic rank of C. sinensis should be reviewed.

When studying the genome and transcriptome of O. felineus, it was found, that expression regulation of almost 50 % of genes is carried out with the participation of trans-splicing machinery (Ershov et al., 2019). This particular form of RNA processing is quite common in flatworms, but the widespread involvement of trans-splicing in trematodes is unusual. For instance, trans-splicing in Schistosoma mansoni is involved in the regulation of transcription of only 11 % of genes in the genome (Protasio et al., 2012).

Trans-splicing is a leader-dependent type of splicing in O. felineus genome. As a result of this process, the 5ʹ-region of the newly synthesized pre-mRNA is replaced by a short sequence of the splice leader encoded by a single gene. In flatworms, this insertion sequence ends in the conservative AUG triplet. It is likely that this triplet can act as a start codon during translation of mature mRNA subjected to trans-splicing. On the other hand, the reason for the expansion of trans-splicing machinery in O. felineus may be its role in the removal of long 5ʹ-non-coding regions from pre-mRNA, which is necessary for the efficient translation of mature transcripts.

Another hypothesis is that trans-splicing is necessary for expression regulation of individual genes in operons. The O. felineus genome revealed 355 potential operons that unite 736 genes separated by trans-splicing sites (Ershov et al., 2019). Predicted operons contain from two to four genes showing different levels of expression. It is possible that the stability of the levels of expression of operon genes is achieved by the fact that the processing of pre-mRNAs synthesized under control of the same promoter is regulated by transsplicing.

Most O. felineus genes, the expression of which is controlled by this mechanism, encode proteins of basic cellular processes (Ershov et al., 2019). Similar data were obtained in the analysis of the Caenorhabditis elegans genome – the most conservative trans-splicing sites were found in the ribosomal genes (Sleumer et al., 2010). An analysis of the genomic data of O. viverrini and C. sinensis also revealed conservative targets for trans-splicing involved in the post-transcriptional regulation of most of the “housekeeping” genes. Obviously, this mechanism plays an important role in the life of flat and round worms, although at present the functional significance of trans-splicing has not been fully understood.

An analysis of the intron lengths in the O. felineus genome revealed (Ershov et al., 2019) that the distribution of the lengths of these elements is characterized by the presence of a large peak at 3000 bp and two additional peaks with maxima at 37 and 90 bp. Ultrashort introns or microintrons less than 75 bp in length make up about 34 % of all annotated introns and are included in the structure of 4997 (44 %) genes. Microintrons are also widely represented in the O. viverrini and C. sinensis genomes. The presence of two peaks of short introns was previously described in tapeworm genomes, and it was assumed that the bimodal distribution of microintrons is a distinctive feature of this group of helminths (Tsai et al., 2013). However, this feature can be traced, albeit less pronouncedly, in the trematodes of the family Opisthorchiidae.

The distribution of microintrons in the genome of O. felineus has some features (Ershov et al., 2019). Firstly, in the presence of several microintrons in a gene, they, as a rule, form clusters. Secondly, microintrons are more often located at the beginning of an exon portion of a gene, i. e. tend to start of gene transcription (Ershov et al., 2019). These facts indicate the separate functional significance of this class of introns in the mechanisms of transcription and processing. Thus, clustering can be associated with recognition of the intron-exon structure by the spliceosome (intron-definition mechanism), and the small size of the microintrons enhances transcriptional efficiency (Urrutia, Hurst, 2003; Belshaw, Bensasson, 2006).

**Mitochondrial genome**

The size of the mitochondrial genome of O. felineus is 13,875 bp, it contains 36 genes: 12 protein-coding genes, two ribosomal RNA genes and 22 transport RNA genes. The mitochondrial genomes of O. felineus, C. sinensis, F. hepatica, and Paragonimus westermani are similarly organized, but differ from the schistosomatid genomes (Shekhovtsov et al., 2010).

## Proteomics and a system of xenobiotic metabolism of O. felineus

The life cycle of the trematode is accompanied by a change in the repertoire of genes expressed at a certain life stage of the parasite. Recently, the results of a comparative study of transcriptomes of metacercariae and adult O. felineus worms (Pomaznoy et al., 2016; Ershov et al., 2019) were published. It was shown, that the transcriptomic profiles of the two life stages of the liver fluke are significantly different: the expression of 903 and 648 genes is registered only in adult or metacercaria, respectively (Pomaznoy et al., 2016). In adult worms, the highest expression is demonstrated for genes encoding proteases, myoglobin, egg shell protein, glutathione S-transferase, and also proteins modulating antigen processing by the host immune cells. In metacercaria, genes encoding “housekeeping” proteins, for example, ribosomal proteins, ubiquitin, and heat shock proteins, have the highest level of expression.

When comparing the transcriptomes of adult O. felineus, O. viverrini and C. sinensis worms, it was found, that the expression levels of the vast majority of genes in the three species of opisthorchids differ slightly (Ershov et al., 2019). This indicates a high similarity in the metabolic processes that ensure the life of helminths in the final host. Nevertheless, the expression of several tens of genes in genomes was species specific. It is important that most of these differentially expressed genes encode the proteins of the excretory secretory product (ESP) of opisthorchids. Species-specific expression of ESP proteins may reflect the host-parasite interaction pecularities.

O. felineus ESPs include various proteins: protective proteins from reactive oxygen species, proteolytic enzymes, carbohydrate metabolism enzymes, protective proteins from the host immune system, cytoskeletal proteins, etc. (Lvova et al., 2014). The one of the major component of the O. felineus ESP is glutathione S-transferase σ (GST-σ). This enzyme retains its activity in an incubation media and is accumulated in the liver tissues of infected animals and patients suffering from opisthorchiasis (Petrenko et al., 2017; Pakharukova et al., 2019). According to a comparative analysis of transcriptomes of adult O. felineus, O. viverrini, and C. sinensis worms, the presence of GST-σ mRNA in the O. felineus transcriptome is many times higher than in the transcriptomes of other opisthorchids. It is likely that this enzyme plays an important role in the host-parasite interaction and can mediate speciesspecific manifestations of the pathogenesis of opisthorchiasis caused by O. felineus. It is important to note that GST-σ might be involved in the metabolism of endogenous substrates and xenobiotics (exogenous substrates), including drugs.

**The O. felineus system of xenobiotic metabolism**

Currently, there are no vaccines or any other means of specific prophylaxis of opisthorchiasis, and the available drugs for chemotherapy of this disease cause complaints (Prichard et al., 2012). In this regard, the study of the xenobiotic metabolism system of liver flukes, the components of which are promising pharmacological targets (Bartley et al., 2012; Prichard et al., 2012), is of particular importance.

With very few exceptions, exogenous substrates that enter living organisms undergo one or more stages of biotransformation, which are carried out by enzymatic biotransformation by means of three phases of metabolism. Phase 1 enzymes, among which the P450 family of proteins (CYPs) are most represented, carry out oxidation, reduction, or hydrolytic reactions of the substrate. An analysis of the available genomic and transcriptomic data of parasitic and free-living flatworms revealed that the composition of CYPs in these groups is markedly different. In free-living species, as in most studied organisms, dozens of weakly homologous to each other diverged CYP genes were found (Table 2). However, parasitic species of the families Opisthorchiidae, Schistosomatidae, Taeniidae, and Fasciolidae own only one cytochrome P450 gene (Pakharukova et al., 2012, 2015). It was shown, that the product of this single gene, CYP in O. felineus liver fluke, is involved in the metabolism of exogenous substrates, is important for the survival of adult worms and represents a promising target for anthelmintic therapy (Pakharukova et al., 2015; Mordvinov et al., 2017b).

**Table 2.**
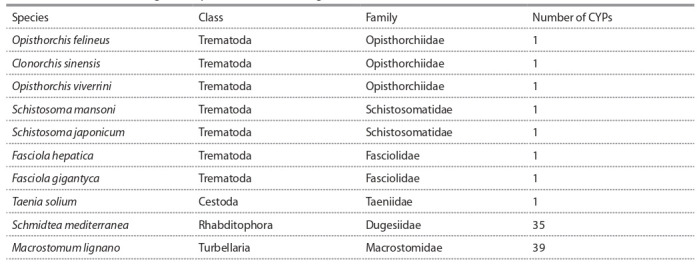
The number of CYP genes in parasitic and free-living flatworms

In addition to CYP gene, other genes encoding phase 1 of xenobiotic biotransformation enzymes were found in the liver fluke genome, in particular, aldo-keto reductase, aldehyde dehydrogenase, and alcohol dehydrogenase genes (Ershov et al., 2019). However, flavin monooxygenase genes, also belonging to phase 1 enzymes, were not found in the O. felineus genome. Interestingly, the sequences of these genes were also not found in the genomic data of other parasitic flatworms.

Glutathione peroxidase and glutathione S-transferase are actively involved in the implementation of phase 2 of xenobiotic metabolism. The O. felineus genome contains nine glutathione S-transferase genes, which are the most highly expressed among all the genes of the xenobiotic metabolism of this liver trematode. The gene encoding GTS-σ is particularly highly expressed, the level of expression of this gene in adult worms is 2–3 orders of magnitude higher than that of other xenobiotic metabolism genes. As already mentioned above, GST-σ is a part of helminth ESP and enters the tissues of infected mammals (Pakharukova et al., 2019). It is important to mention here that, in addition to the transferase activity, GST-σ has the properties of prostaglandin synthase, is involved in the production of prostaglandins, and might retain this enzymatic activity in the host tissues (Morphew et al., 2007).

Another group of enzymes that are usually involved in the implementation of phase 2 metabolism of xenobiotics in eukaryotes is UDF-glucuronyl transferase (UGT). The functions of these proteins are to increase the hydrophilicity of substrates and their availability for the cell excretion, phase 3. The superfamily of UGTs consists of two families of UGT1 and UGT2, combining more than 20 isoenzymes. In the genomes of parasitic and free-living nematodes, from 30 to 70 genes encoding UGT were found (Matouskova et al., 2016). These enzymes play an important role in the formation of parasite resistance to anthelmintics (Lindblom et al., 2006; Laing et al., 2013; Matouskova et al., 2016). However, neither the genome of O. felineus, nor the genomes of other representatives of the Opisthorchiidae family, trematodes of the Schistosomatidae family, contained UGT genes. In addition, genes encoding arylamine N-acetyltransferase, enzymes involved in phase 2 reactions of xenobiotic metabolism in vertebrates were not found in the opisthorchid and schistostomatid genomes (Ershov et al., 2019).

Proteins of phase 3 of xenobiotic metabolism are responsible for excretion from cells into the extracellular space of compounds formed as a result of the action of phase 1 and 2 enzymes. Excretion is carried out by proteins belonging to five families of membrane transporters. ABC transporters are the best studied phase 3 proteins, since these proteins are involved in the drug resistance mechanisms of eukaryotic and prokaryotic cells (Saier et al., 2014; Wong et al., 2014). Twenty-three genes encoding ABC transporters were found in the O. felineus genome (Mordvinov et al., 2017a). Interestingly, four of them, P1–P4, are similar to the single human P-glycoprotein gene. The product of this gene is also known as multidrug resistance protein 1 (Saier et al., 2014; Wong et al., 2014). It was found that in two O. felineus P-glycoprotein genes, the expression level depends on the stage of development of the parasite. So, in adult worms, the expression of the P1 and P4 genes is 20–30 times higher than in metacercariae and recently excised juvenile worms. It is probably, that these proteins are most significant for the metabolism of xenobiotics in adult parasites.

In conclusion, it should be emphasized that the O. felineus xenobiotic metabolism system, as, probably, in other parasitic flatworms, has clear structural and functional features. First, it differs significantly from the xenobiotic metabolism system of the mammalian hosts. A detailed study of the xenobiotic metabolism system of the liver flukes will expand our understanding of the development of parasitism mechanisms and the evolution of host–parasite relationship. Knowledge of the structure and functions of this metabolic system can also be applied in the identification of new pharmacological targets for the treatment of opisthorchiasis and other trematodiases.

The products of the xenobiotic metabolism system of O. felineus and other trematodes can be metabolites presented in the ESP of parasites. Low molecular weight components of O. felineus ESP, parasite-specific cholesterol metabolites were found (Gouveia et al., 2017). These oxysterol-like compounds possess genotoxic properties and might cause damage to the host DNA. The accumulation of such damage leads to malignant transformation of bile duct tissue. It is possible that specific O. felineus oxysterols are involved in triggering cholangiocarcinogenesis mechanisms during opisthorchiasis.

The synthesis of parasite-specific oxysterols can be carried out by CYP and other redox enzymes, such as glutathione S-transferase, thioredoxin peroxidase, etc. The search for proteins involved in the enzymatic pathway for the generation of specific genotoxic helminthic oxysterols remains a priority for molecular parasitology.

**Granulins as potential promoters of cholangiocyte neoplasia**

There is a hypothesis that granulin, a protein that is part of the opisthorchid ESP, participate in carcinogenic processes associated with helminth infection (Smout et al., 2015). Granulins of O. felineus, O. viverrini and C. sinensis liver flukes have conserved structure and are homologous to human granulin. Human and helminthic granulins stimulate the proliferation of epithelial cells, including cholangiocytes (Smout et al., 2015), however, they use various cellular signaling pathways. Human granulin acts as an antagonist of the tumor necrosis factor (TNF) signaling pathway. The helminthic granulin receptor is unknown, but it was found that O. viverrini granulin enters into cholangiocytes and activates the MAP kinase and epidermal growth factor receptor signaling pathway. This is a much more powerful way to activate proliferation than that used by human granulin.

O. viverrini granulin effectively promotes the healing of injuries (Smout et al., 2015). In addition, this protein stimulates the growth of blood vessels (Smout et al., 2015) and can probably activate cell migration. It is believed, that granulin facilitates the proliferation of malignant cholangiocytes arising from chronic opisthorchiasis, and contributes to the development of a bile duct cancer.

In the O. felineus genome, as in the O. viverrini and C. sinensis genomes, four genes (GRN-1–GRN-4) encoding singledomain granulins, as well as one multi-domain progranulin (PGRN) gene were found (Ershov et al., 2019). Genes of single-domain granulins are localized in one chromosomal locus and form a conservative syntenic group of genes. The sequences of the GRN-1 and GRN-4 O. felineus genes have 95 % homology, which suggests the duplication of a single gene. The fixation of this duplication in the genomes of opisthorchids can probably be associated with a significant functionality of granulin.

The highest level of expression of the O. felineus GRN-1 and GRN-4 genes was shown in adult helminths, while in metacercaria the GRN-3 gene is dominantly expressed (Ershov et al., 2019). All experimental work on the determination of the potentially carcinogenic properties of opisthorchid granulins was performed with the O. viverrini GRN-1. It can be assumed that the product of the O. felineus GRN-4 gene also has mitogenic, angiogenic properties and the ability to increase cell migration. In adults, expression of the GRN-2 and GRN-3 genes is practically absent. It is likely that the products of these genes may be involved in the host–parasite relationship in intermediate hosts of trematodes, mollusks and fish.

## Conclusion

The causative agent of opisthorchiasis, the O. felineus liver fluke is epidemiologically significant species of Opisthorchiidae trematodes. Its range covers vast areas of Europe and Asia, and outbreaks of opisthorchiasis caused by this helminth can be expected in many countries. One cannot but take into account the growing migration of the population and the tourist flow between different countries. Due to these factors, patients suffering from O. felineus infection can be detected far beyond the endemic areas. Thus, opisthorchiasis caused by O. felineus infection becomes a global challenge that goes beyond the biomedical problems of individual regions.

The appearance of the genomic and proteomic data of O. felineus significantly strengthens the basis of molecular biological studies of epidemiologically important liver flukes. In-depth studies of the genomics and proteomics of O. felineus will allow the generation of substantiated hypotheses about the carcinogenesis mechanisms associated with opisthorchiasis, the identification of species-specific pathogenesis of helminthiases, and the targeted search for molecular targets for the treatment of these diseases. The research strategies should consider the urgent need of practical health care for effective means of therapy and prevention of trematodiases.

## Conflict of interest

The authors declare no conflict of interest.
